# *Candida albicans* Induces Foaming and Inflammation in Macrophages through FABP4: Its Implication for Atherosclerosis

**DOI:** 10.3390/biomedicines9111567

**Published:** 2021-10-29

**Authors:** Mohammed Haider, Fatema Al-Rashed, Zahraa Albaqsumi, Khaled Alobaid, Rawan Alqabandi, Fahd Al-Mulla, Rasheed Ahmad

**Affiliations:** 1 Department of Biological Sciences, Faculty of Science, Kuwait University, Kuwait City 15462, Kuwait; mohammed.haider@ku.edu.kw; 2 Immunology & Microbiology Department, Dasman Diabetes Institute, Kuwait City 15462, Kuwait; fatema.alrashed@dasmaninstitute.org (F.A.-R.); zahraa.albaqsumi@dasmaninstitute.org (Z.A.); rawan.alqabandi@dasmaninstitute.org (R.A.); 3 Mycology Reference Laboratory, Medical Laboratory Department, Mubarak Al-Kabeer Hospital, Kuwait City 15462, Kuwait; khaled22m@live.com; 4 Genetics & Bioinformatics, Dasman Diabetes Institute, Dasman 15462, Kuwait; fahd.almulla@dasmaninstitute.org

**Keywords:** *C. albicans*, macrophages, fat accumulation, inflammation, MMP-9, FABP4

## Abstract

Atherosclerosis is a chronic degenerative disorder characterized by lipid-dense plaques and low-grade inflammation affecting arterial walls. Foamy macrophages are important in the formation of atherosclerotic plaques and the induction of low-grade inflammation. The presence of lipid-laden macrophages has occurred in infections caused by opportunistic pathogens. *Candida albicans* is the major cause of candidiasis in immunocompromised patients, including those with diabetes mellitus. However, the role played by *C. albicans* in macrophage foaming and the associated inflammation is poorly understood. We investigated whether *C. albicans* induces foaming along with inflammation in macrophages and, if so, by which mechanism(s). We incubated THP-1 macrophages with heat-killed *C. albicans* (HKCA). HKCA-induced lipid accumulation in macrophages along with increased expression of inflammatory markers, including CD11b and CD11c or expression and secretion of IL-1β. HKCA also increased the expression of PPARγ, CD36, and FABP4 in macrophages. Mechanistically, we found that the foamy and inflammatory macrophage phenotype induced by HKCA requires FABP4 because disruption of FABP4 in macrophages either by chemical inhibitor BMS309404 or small interfering RNA (siRNA) abrogated foam cell formation and expression of inflammatory markers CD11b, CD11c, and IL-1β. Furthermore, HKCA-treated macrophages displayed high expression and secretion of MMP-9. Inhibition of FABP4 resulted in suppression of HCKA-induced MMP-9 production. Overall, our results demonstrate that *C. albicans* induces foam cell formation, inflammation, and MMP-9 expression in macrophages via the upregulation of FABP4, which may constitute a novel therapeutic target for treating *C. albicans*-induced atherosclerosis.

## 1. Introduction

Atherosclerosis is one of many diseases that are associated with a high mortality rate. Its pathological etiology remains unclear because of its multifactorial risk factors, including diabetes mellitus, smoking, obesity, and arterial hypertension [1,2,3]. Studies have shown that the damage caused by atherosclerosis is inflammation-based due to the formation and rupturing of vascular plaques that are characterized by excessive amount of foamy macrophages and depletion of collagen. Furthermore, the pathology is emphasized by proteolytic degradation of the extracellular matrix (ECM) [4,5]. In atherosclerosis, excessive matrix proteolysis mediated by matrix metalloproteinases (MMPs), particularly MMP-9, is thought to be a common and important stage in the development of lesions [6].

Several studies have reported a correlation between specific pathogenic infections, such as chlamydia, pneumonia, tuberculosis, and lipid accumulation in association with atherothrombosis [4,7]. Few researchers have investigated the role of opportunistic fungi in the etiology of atherosclerosis [7]. *C. albicans* is an opportunistic pathogen and is a natural inhabitant of the human oral, intestinal, and vaginal tracts. It can cause infections ranging from mild illness to systemic, life-threatening candidiasis [2,8]. *C. albicans* induces inflammation in the vascular wall [8,9]. Macrophages play a major role in the inflammatory progression observed in atherosclerosis. They are recruited in response to the release of pro-inflammatory signals, including TNF-α and IL-6 [1,10,11]. In the presence of pathogen-derived agonists, lipid bodies were shown to form and trigger the formation of foamy macrophages [12]. Other studies have suggested that this process is induced by imbalanced influx and efflux of low-density lipoprotein (LDL) particles from serum [13].

Herein, we investigated whether *C. albicans* induces foaming along with inflammation in macrophages and, if so, by which mechanism(s). In this study, we show for the first time that stimulation with HKCA induces foaming and inflammation in mac-prophages. HKCA-treated macrophages showed high levels of fat accumulation with increased expression of the pro-inflammatory markers CD11b and CD11c, and secretion of IL-1β and MMP-9. Mechanistically, we found that the foamy and inflammatory macrophage phenotype induced by HKCA requires FABP4.

## 2. Materials and Methods

### 2.1. Cell Culture

Human monocytic THP-1 cells were purchased from American Type Culture Collection (ATCC) and grown in RPMI-1640 culture medium (Gibco, Life Technologies, Grand Island, NY, USA) supplemented with 10% fetal bovine serum (Gibco), 2 mM glutamine (Gibco), 1 mM sodium pyruvate, 10 mM HEPES, 100 ug/mL Normocin, 50 U/mL penicillin, and 50 μg/mL streptomycin (P/S; Gibco) [14]. Cells were incubated at 37 °C with humidity in 5% CO_2_. 

### 2.2. Macrophage Differentiation

THP-1 cells were differentiated into macrophages as previously described [15,16]. In brief, THP-1 cells were treated with 10 ng/mL PMA (Phorbol 12-myristate 13-acetate) for three days in in RPMI media supplemented with 10% fetal bovine serum (Gibco, Life Technologies, Grand Island, NY, USA), 2 mM glutamine (Gibco, Invitrogen, Grand Island, NY, USA), 1 mM sodium pyruvate, 10 mM HEPES, 100 ug/mL Normocin, 50 U/mL penicillin, and 50 μg/mL streptomycin (Gibco, Invitrogen, Grand Island, NY, USA). Adherent cells were then washed and incubated in serum-free RPMI media for a further three days before they were considered ready for treatment.

### 2.3. Generation of Heat-Killed C. albicans

*C. albicans* was a kind gift from Dr. Khaled Alobaid (Mycology Reference Laboratory at Mubarak Al-Kabeer Hospital, Kuwait). Single colonies of the clinical isolate (141/11/20) were inoculated into yeast extract peptone dextrose (YPD) broth and incubated at 30 °C for 48 h, with shaking at 200 rpm. YPD broth was made by dissolving 10 g yeast extract (Oxoid, Cambridge, UK), 20 g mycological peptone (Oxoid), and 20 g D-glucose (Fisher Scientific, Loughborough, UK) in 1 L distilled water and then autoclaving the mixture. The cultures were then centrifuged three times at 6000 rpm in an Eppendorf 5415 R centrifuge (Eppendorf^®^, Hamburg, Germany), resuspended in PBS, and adjusted to a final concentration of 1 × 10^8^/mL. Heat-killing was then done by incubating cells at 100 °C for 1 h on a static heat block. Killing was confirmed by plating out cells on YPD agar and monitoring growth for up to 72 h. YPD agar was prepared like that used for the YPD broth described above but also included 20 g technical agar (Oxoid) for every 1 L preparation.

### 2.4. Cell Stimulation

Monocytes were plated in 12-well plates (Costar, Corning Incorporated, Corning, NY, USA) at 1 × 10^6^ cells/well unless indicated otherwise. Cells were differentiated into macrophages, as described previously, and then pretreated with FABP4 inhibitor (BMS309403) (Sigma BM0015; 25 µM) for one hour. Cells were then stimulated with HKCA at a ratio of 10:1 fungus: macrophage, lipopolysaccharide (LPS) (10 ng/mL; L4391, Sigma Aldrich, Merck KGaA, Darmstadt, Germany) or 0.01% DMSO (vehicle control) overnight at 37 °C. In order to assess CD36 gain/loss-of-function, cells were treated with the CD36 agonist Rosiglitazone at a concentration of 1.5 µM, or 250 µM of the CD36 inhibitor Sulfosuccinimidyl oleate (SSO) for one hour, followed by overnight stimulation with HKCA. Cells were harvested for either RNA or protein analysis. Culture media were collected to analyze IL-1β secretion.

### 2.5. Quantitative Real-Time PCR

Total RNA was extracted using RNeasy Mini Kit (Qiagen, Valencia, CA, USA) per the manufacturer’s instructions. cDNA was synthesized from 1 μg of total RNA using high-capacity cDNA Reverse Transcription Kits (Applied Biosystems, Foster City, CA, USA) [17,18]. Quantitative real-time PCR (qRT-PCR) was performed on a 7500 Fast Real-Time PCR System (Applied Biosystems) using TaqMan^®^ Gene Expression Master Mix (Applied Biosystems). Each reaction contained 50 ng of cDNA amplified with inventoried TaqMan Gene Expression Assay products (CD36: Assay Hs00354519_m1; FABP4: Assay Hs01086177_m1; MMP-9: Assay ID: Hs00957562_m1; GAPDH: Hs03929097_g1). The threshold cycle (*Ct*) values were normalized to the housekeeping gene GAPDH, and the amounts of target mRNA relative to control were calculated using the ΔΔ*Ct* method [19,20]. Relative mRNA expression was expressed as fold expression over the average of control gene expression. The expression level in the control treatment was 1 [21]. Values are presented as mean ± SEM. Results were analyzed statistically; *p* < 0.05 was considered to indicate significant differences.

### 2.6. Flow Cytometry: Staining of Cell-Surface Markers

Monocytic cells were seeded in 24-well plates at 5 × 10^5^ cell/mL and then transformed into macrophages, as described previously. The cells were treated with the FABP4 inhibitor BMS309403 (25 µM) or 0.01% DMSO (vehicle) for one hour and then subjected to overnight stimulation with HKCA at a ratio of 10:1 fungus: macrophage or PBS (vehicle). The macrophages were then washed in ice-cold PBS to allow detachment, resuspended in FACS staining buffer (BD Biosciences), and blocked with human IgG (Sigma; 20 μg) for 30 min on ice. The cells were washed and resuspended in 100 µL FACS buffer and incubated with anti-CD11c PE-Cy7 (cat # 117317; BD Biosciences), anti-CD11b (D12)-APC (cat # 340936; BD Biosciences), anti-CD36-FITC (cat # 555454; BD Pharmingen™), or suitable isotype control antibody (Cat # 558055; BD Phosflow™, Cat # 559529; BD Phosflow™, Cat # 560542; BD Pharmingen™; or Cat # 560817; BD Phosflow™) on ice for 30 min. BODIPY 495/503 stain (Cat # D3922, Life Technologies) was used to quantify lipid levels. The cells were washed three times with FACS buffer and resuspended in 2% paraformaldehyde. The cells were centrifuged and resuspended in FACS buffer for FACS analysis (FACSCanto II; BD Bioscience, San Jose, CA, USA). FACS data analysis was performed using BD FACSDivaTM Software 8 (BD Biosciences) [15,22]. Unstained cells were used to set the quadrant of the negative vs. positive gates. The stain index (SI) was calculated as the difference between the mean fluorescence intensity of the positive and negative populations, divided by two times the standard deviation of the negative populations (unstained cells).

### 2.7. Flow Cytometry: Intracellular Staining

Flow cytometric analysis was used to investigate the expression of intracellular IL-1β. Briefly, cells were stained for extracellular markers as described above. The cells were then incubated with fixation/permeabilization buffer (cat# 00-5523-00, eBioscience, San Diego, CA, USA) for 20 min at 4 °C, followed by washing and staining with mouse anti-human IL-1β- PE (cat # 340516; BD Biosciences) for 30 min. The cells were then washed and resuspended in PBS supplemented with 2% FCS for FACS analysis (FACSCanto II; BD Bioscience). FACS data analysis was performed using BD FACSDiva^TM^ Software 8 (BD Biosciences).

### 2.8. Sandwich Enzyme-Linked Immunosorbent Assay

Macrophages were cultured as described previously, and the culture medium was harvested 24 h post-stimulation/incubation. The IL-1β protein in the supernatants was quantified using sandwich enzyme-linked immunosorbent assay (ELISA) following the manufacturer’s instructions (R&D Systems, Minneapolis, MN, USA).

### 2.9. Small Interfering RNA Transfection

Monocytes were washed and resuspended in 100 µL of the Nucleofector solution provided with the Amaxa Nucleofector Kit V (Lonza, Germany), and transfected separately with siRNA-FABP4 (30 nM; OriGene Technologies Inc., Rockville, MD, USA) or scrambled (control) siRNA (30 nM; OriGene Technologies, Inc., Rockville, MD, USA), and pmaxGFP (0.5 µg; Amaxa Noclecfector Kit V for THP-1, Lonza). All transfection experiments were performed using the Amaxa Cell Line Nucleofector Kit V for monocytic cells (Lonza) using an Amaxa Electroporation System (Amaxa Inc., Cologne, Germany) according to the manufacturer’s protocol [23]. After 36 h of transfection, cells were cultured as described previously. The level of FABP4 gene knockdown was assessed using western blotting and qRT-PCR.

### 2.10. Nile Red Staining of Lipids

Accumulated neutral lipids were visualized using Nile Red fluorescence staining (Sigma Aldrich, Germany). THP-1 cells were plated on coverslips and allowed to rest for four hours. Cells were then differentiated into macrophages and treated as described previously. Cells were then treated with 4% paraformaldehyde for 20 min followed by three washes in PBS, after which they were considered ready for staining. Briefly, cells were incubated with Nile Red (1 µM in PBS) for 10 min at ambient temperature, protected from light. After extensive washing, the cells were counterstained and mounted on slides using a mountant containing DAPI (Vectashield, Vectorlab, H1500). Confocal images were obtained on an inverted Zeiss LSM710 spectral confocal microscope (Carl Zeiss, Gottingen, Germany) with an EC Plan-Neofluar 40×/1.30 oil DIC M27 objective lens using a Green Fluorescent Protein filter (λEx: 450–500 nm; λEm: 528 nm) and a DAPI-filter (λEx: 395 nm; λEm: 460 nm). All samples were analyzed using the same parameters, and the resulting color markup of analysis was confirmed for each sample.

### 2.11. Western Blotting

Macrophages were harvested and incubated for 30 min with lysis buffer (Tris 62.5 mM, pH 7.5, 1% Triton X-100, 10% glycerol). The lysates were centrifuged at 14,000× *g* for 10 min, and the supernatants were collected. The concentrations of proteins in the lysates were measured using Quickstart Bradford Dye Reagent, 1× Protein Assay kits (Bio-Rad Laboratories, Inc, Hercules, CA, USA). Protein samples (20 µg) were mixed with sample loading buffer, heated for 5 min at 95 °C, and resolved on 12% polyacrylamide gels using SDS-PAGE. Cellular proteins were transferred to Immuno-Blot PVDF membranes (Bio-Rad Laboratories) by electroblotting. The membranes were then blocked with 5% non-fat milk in PBS for 1 h followed by incubation with primary antibodies against CD36 (MW: 70–110 kDa), FABP4 (MW: 15 kDa), or β-actin (MW: 42 kDa) at a 1:1000 dilution at 4 °C overnight. All primary antibodies were purchased from Cell Signaling (Cell Signaling Technology, Inc., Danvers, MA, USA). The blots were then washed four times with TBS and incubated for 2 h with HRP-conjugated secondary antibody (Promega, Madison, WI, USA). Immunoreactive bands were developed using an Amersham ECL Plus Western Blotting Detection System (GE Health Care, Buckinghamshire, UK) and visualized using a Molecular Imager^®^ ChemiDocTM MP Imaging Systems (Bio-Rad Laboratories, Hercules, CA, USA) [24].

### 2.12. Statistical Analysis

Data are shown as mean ± SEM, and statistical analysis was performed using Prism 8.3.1 software (GraphPad Inc., San Diego, CA, USA). Group means were compared using one-way analysis of variance followed by Tukey’s post hoc tests. All *p*-values ≤ 0.05 were considered to indicate statistical significance.

## 3. Results

### 3.1. HKCA Stimulation Induced Foaming and Inflammation in Human Macrophages

**Figure 1:**
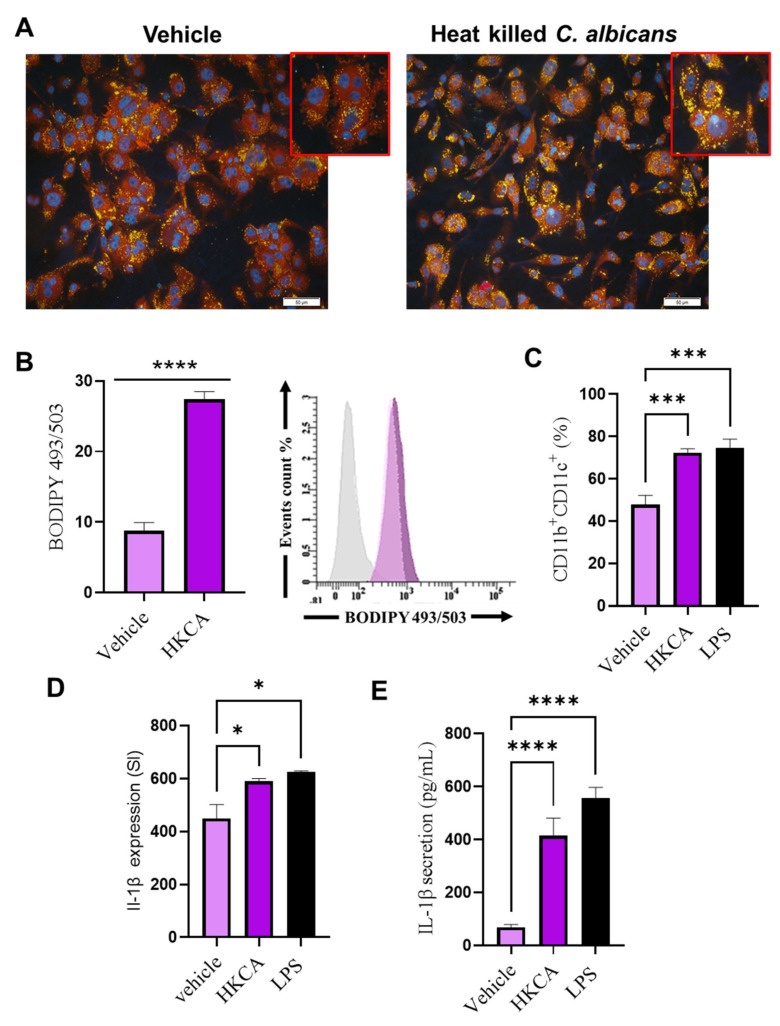
Stimulation with HKCA induces macrophage foaming. Differentiated human THP-1 macrophages were stimulated overnight with heat-killed *Candida albicans* (HKCA), and the level of intracellular lipids was assessed using (**A**) Nile red fluorescence staining. (**B**) Bar graph of mean staining index (SI) of BODIPY 493/503 calculated from three independent determinations, with the similar result presented in the histogram. (**C**) HKCA and LPS (a positive control for inflammation)-treated cells were labeled with the macrophage proinflammatory surface markers CD11b and CD11c. (**D**) CD11b^+^CD11c^+^ cells were labeled for intercellular IL-1β expression. (**E**) IL-1β protein secretion was measured in the media using an enzyme-linked immunosorbent assay. Results were obtained from three independent experiments. All data are expressed as mean ± SEM values of triplicate samples (*n* = 3). * *p* ≤ 0.5, *** *p* ≤ 0.001, **** *p* ≤ 0.0001 and ns: non-significant. Images are shown at 20× magnification; Scale bar = 50 µm.

To test whether stimulation by *C. albicans* induced cell foaming, we incubated differentiated THP-1 human macrophages with HKCA overnight. We investigated the level of lipid droplet formation using the fluorescent dye Nile Red to distinguish between normal cells and cells containing higher levels of triglycerides (neutral lipids) [25]. Using confocal microscopy analysis, revealed higher levels of neutral lipid droplets that fluoresce a brilliant yellow-gold in HKCA-treated macrophages compared with vehicle controls (Figure 1A). These results were confirmed using flow cytometric analysis of BODIPY 493/505 (Figure 1B). We found a significant accumulation of lipids within HKCA-treated macrophages (*p* < 0.00001), these findings suggest that stimulation with HKCA induces lipid accumulation in differentiated macrophages. We next investigated whether HKCA could augment the cell surface expression of M1 proinflammatory markers. To test this, macrophages were treated with Vehicle, HKCA, or LPS (as a positive control for inflammation). We found that the stimulation with HKCA significantly increased a population of cells that are double-positive for the M1 proinflammatory markers CD11b and CD11c (CD11b^+^CD11c^+^ cells), as observed when cells were treated with LPS, a positive control for inflammation (Figure 1C). Macrophages expressing CD11b^+^CD11c^+^ on their surface had higher levels of IL-1β intracellular cytokine production (Figure 1D). IL-1β secretion was elevated in HKCA-stimulated cultures (Figure 1E). However, we did not see any upregulation of TNF-α or MCP-1 in macrophages treated with HKCA (Supplementary Figure S1A,B). Together, these data indicate that stimulation by *C. albicans* triggers the production of lipid-laden macrophages that exhibit proinflammatory properties.

### 3.2. HKCA Stimulation Upregulates the Expression of CD36 and FABP4 in Foamy Macrophages

**Figure 2:**
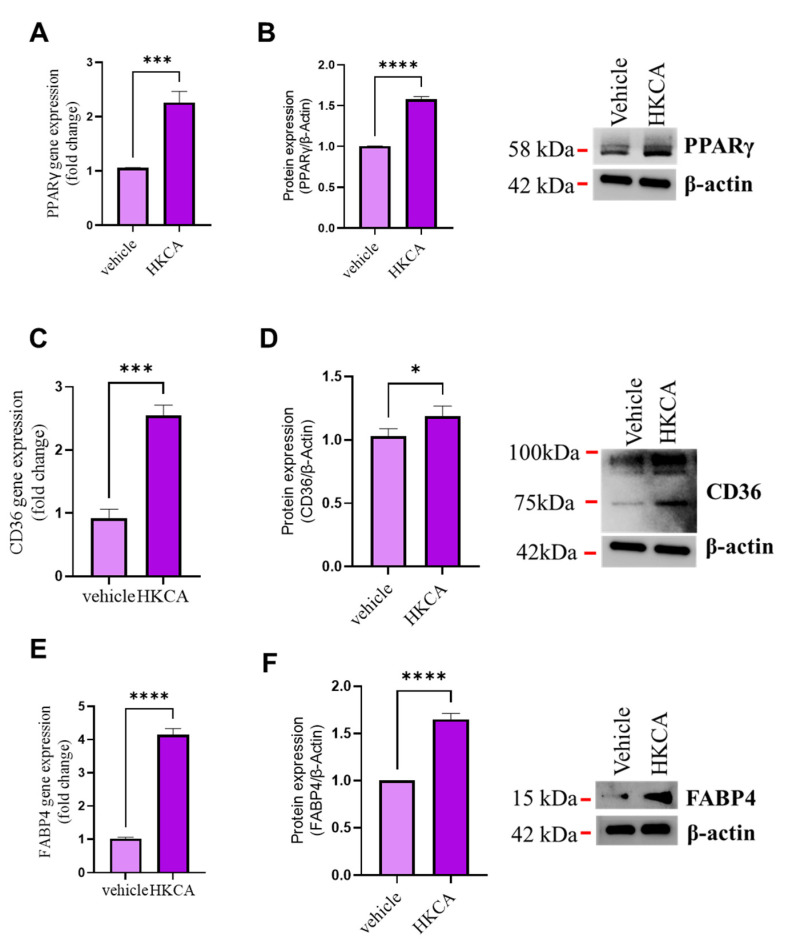
HKCA stimulation regulates the expression of genes involved in macrophage foam cell formation. Differentiated macrophages were stimulated by heat-killed *Candida albicans* (HKCA). Gene and protein expression levels were measured using qRT-PCR and Western blotting, respectively. (**A**,**B**) Peroxisome proliferator-activated receptor gamma (PPARγ), (**C**,**D**) CD36, and (**E**,**F**) Fatty Acid Binding protein 4 (FABP4). The results were obtained from three independent experiments. All data are expressed as mean ± SEM values of triplicate samples (*n* = 3). * *p* ≤ 0.05, *** *p* ≤ 0.001 and **** *p* ≤ 0.0001.

The expression of CD36 and FABP4 has previously been shown to facilitate macrophage/foam cell formation, as both act as molecular targets of PPARγ and have been implicated in atherosclerotic plaque formation [26,27]. To determine whether *C. albicans* exerts any modulatory effects on PPARγ and its target genes CD36 and FABP4 in vitro, we incubated macrophages with HKCA or vehicle control. Overnight incubation of macrophages with HKCA was found to lead to significant upregulation of the expression of PPARγ at both the gene (Figure 2A) and protein levels (Figure 2B). HKCA stimulation induced the expression of two PPARγ reporters: the scavenger receptor CD36 (Figure 2C,D) and Fatty Acid Binding Protein 4 (FABP4) (Figure 2E,F), with a stronger effect observed for the latter. These results indicate that *C. albicans* triggers macrophage foaming, and this effect is associated with the upregulation of the lipid uptake markers CD36 and FABP4, which might be driven by the action of PPARγ.

### 3.3. FABP4 Inhibition Prevents HKCA-Induced Macrophage Foaming and Associated Inflammation

**Figure 3:**
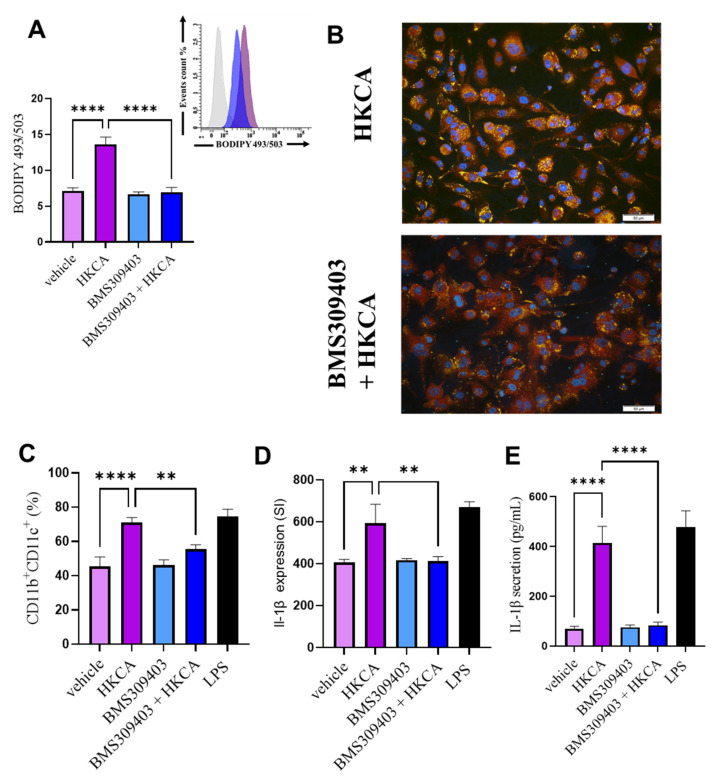
FABP4 inhibition prevents HKCA-induced macrophage foaming. Differentiated macrophages were pretreated with either Fatty Acid Binding Protein 4 (FABP4) inhibitor (BMS309404; 25 µM) or vehicle (dimethylsulfoxide; DMSO) for one hour followed by overnight stimulation with heat-killed *Candida albicans* (HKCA). Intracellular lipid accumulation was assessed by (**A**) flow cytometric analysis of BODIPY 493/503 presented in a bar graph and histogram representative of three independent experiments. (**B**) Nile Red immunofluorescence staining of lipids. Images are shown at 20× magnification; Scale bar = 50 µm. The macrophage proinflammatory phenotype was determined by measuring (**C**) surface expression of CD11b and CD11c, (**D**) intracellular expression of IL-1β in CD11b^+^CD11c^+^ cells. (**E**) IL-1β protein secretion was measured by enzyme-linked immunosorbent assay. The results were obtained from three independent experiments. All data are expressed as mean ± SEM of triplicate samples (*n* = 3). ** *p* ≤ 0.01, and **** *p* ≤ 0.0001.

FABP4 plays a critical role in regulating lipid metabolism and inflammation [28,29]. To assess the potential involvement of FABP4 in HKCA-mediated macrophage foaming, we treated differentiated macrophages with either vehicle or the FABP4 inhibitor BMS309403 [30] before their stimulation with HKCA. Macrophages pretreated with BMS309404 accumulated significantly fewer intracellular lipid droplets, as observed using flow cytometric analysis of BODIPY 493/505 and Nile Red immunostaining (Figure 3A,B, respectively). FABP4 inhibition was also associated with significantly reduced populations of CD11b^+^CD11c^+^ cells, compared with cells stimulated with HKCA in the absence of the inhibitor (Figure 3C). FABP4 inhibition was also associated with a significant reduction in the gene expression and protein secretion of IL-1β compared with cells stimulated with HKCA in the absence of the inhibitor (Figure 3D,E). Furthermore, our data show that the inhibition of CD36 suppress HKCA-mediated lipid accumulation and inflammation in macrophages (Supplementary Figure S2A,D).

### 3.4. FABP4 Deficiency Suppresses HKCA-Induced Macrophage Foaming and Inflammation

**Figure 4:**
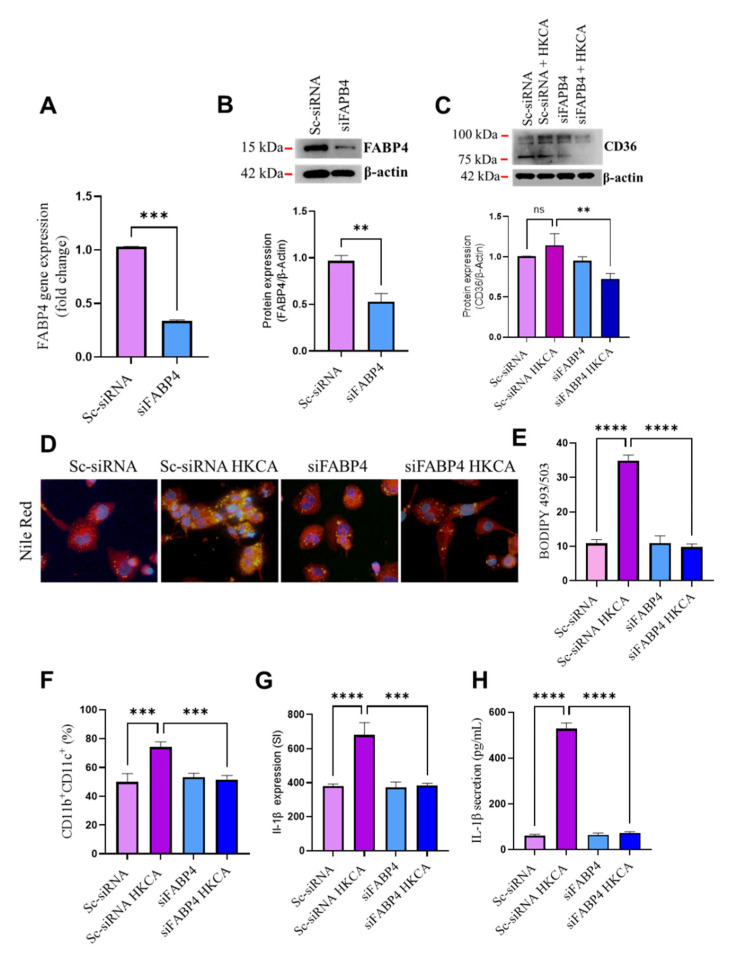
FABP4 deficiency abolishes macrophage foaming and inflammation induced by HKCA. Monocytes were transfected with scrambled siRNA (mock/negative control) or Fatty Acid Binding protein 4 (FAPB4) siRNA and incubated for 36 h to induce transformation into macrophages. Differentiated FABP4-deficient cells were stimulated with heat-killed *Candida albicans* (HKCA). (**A**) FABP4 gene expression in transfected cells was measured using qRT-PCR. (**B**) FABP4 protein expression in transfected cells was measured using Western blotting. (**C**) Upstream protein expression of CD36 was measured in transfected cells using Western blotting. (**D**) Intracellular lipid accumulation was assessed by Nile Red immunofluorescence staining of lipids and (**E**) BODIPY 493/503. The macrophage proinflammatory phenotype was determined by measuring (**F**) the surface expression of CD11b and CD11c. (**G**) The intracellular expression of IL-1β in CD11b^+^CD11c^+^ cells. (**H**) IL-1β protein secretion was measured in media using enzyme-linked immunosorbent assay (ELISA). The results were obtained from three independent experiments. All data are expressed as mean ± SEM of triplicate samples (*n* = 3). ** *p* ≤ 0.01, *** *p* ≤ 0.001, and **** *p* ≤ 0.0001, ns: non-significant. Images are shown at 40× magnification; Scale bar = 20 µM.

To further verify that HKCA-induced macrophage foaming is FABP4-dependent, we transfected cells with siRNA against FABP4 (siFABP4), which produced a more than 75% reduction in FABP4 mRNA levels, and reduced protein levels of ~50% compared with scrambled (control) siRNA (Sc-siRNA) (Figure 4A,B). CD36 protein expression upstream of FABP4 was lower in FABP4-deficient cells after HKCA stimulation (Figure 4C), indicating a modulatory role for FABP4 in the function of CD36. As in our previous observations, macrophage foaming (Figure 4D,E) and M1 proinflammatory markers after HKCA stimulation were both suppressed in FABP4-deficient cells (Figure 4F–H).

### 3.5. Effect of FABP4 Inhibition on HKCA-Induced Production of MMP-9

**Figure 5:**
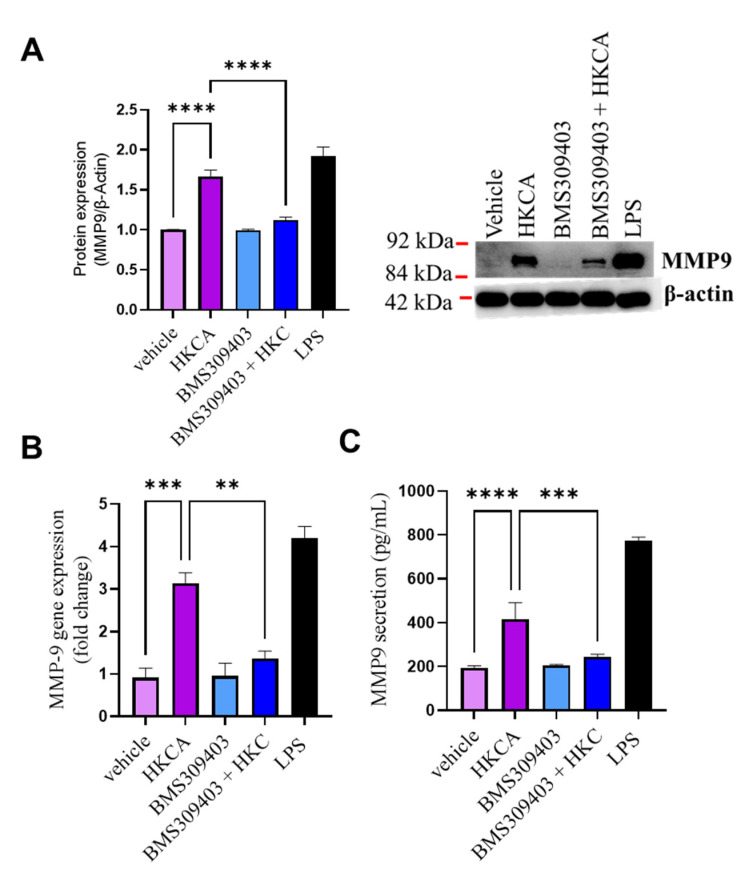
Effect of FABP4 inhibition on MMP-9. Differentiated macrophages were pretreated with either Fatty Acid Binding Protein 4 (FABP4) inhibitor (BMS309404) or vehicle (DMSO) for one hour followed by overnight stimulation with heat-killed *Candida albicans* (HKCA). LPS treatment was used as a positive control for MMP-9 production. (**A**) Matrix metallopeptidase 9 (MMP-9) protein expression was determined by Western blotting. Data are representative of three independent experiments. (**B**) MMP-9 gene expression was measured using qRT-PCR as described in the Materials and Methods section. (**C**) MMP-9 secreted protein in culture supernatants was determined by enzyme-linked immunosorbent assay (ELISA). The results were obtained from three independent experiments. All data are expressed as mean ± SEM of triplicate samples (*n* = 3). ** *p* ≤ 0.01, *** *p* ≤ 0.001, and **** *p* ≤ 0.0001.

Increases in circulating levels of MMP-9 occur in several metabolic syndromes such as type 2 diabetes (T2D) and cardiovascular diseases such as atherosclerosis [31,32]. MMP-9 activity in macrophages induced foamy macrophage migration and proliferation into cardiac smooth muscle cells during the early stages of the atherosclerotic process [33]. In our experimental setting, HKCA stimulation triggered macrophage foaming. Therefore, we questioned whether HKCA stimulation of macrophages triggered MMP-9 production and whether FABP4 inhibition could prevent its expression. Macrophages stimulated with HKCA were found to significantly upregulate MMP-9 expression at both the gene and protein levels. Inhibition by the FABP4 inhibitor BMS309403 significantly blocked the HKCA-induced gene expression of MMP-9 (Figure 5A,B). HKCA stimulation also induced MMP-9 secretion in culture media. This effect was reduced in FABP4-inhibited cells (Figure 5C). We found similar results when we inhibited CD36 upstream of FABP4 (Supplementary Figure S3).

## 4. Discussion

Several recent studies have highlighted the influence of some infectious pathogens, including *Porphyromonas gingivalis* and *Chlamydia pneumoniae*, on the development of atherosclerosis [34,35]. Atherosclerosis is a chronic, lipid-driven inflammatory disease of the blood vessel walls, caused primarily by an innate immune response. A pivotal role in the pathogenesis of atherosclerosis is played by foamy macrophages that reside in the intima aortic wall, forming atherosclerotic plaques. Although a relationship between the destabilized atherosclerotic process and fungal infections has been reported [8,36,37], most studies have focused on viral and bacterial infections, with only a few examining the role of fungal pathogens in this process. This is the first study to report that HKCA stimulation induces lipid accumulation in macrophages to the best of our knowledge. We found that HKCA stimulation of macrophages in the presence of serum-rich media-induced foam cell formation. Foam cell formation in response to HKCA initiated a proinflammatory response, as evinced by the upregulated macrophage M1 proinflammatory cell surface markers CD11b and CD11c and the downstream expression and secretion of the pro-inflammatory cytokine IL-1β. The expression of CD11b and CD11c has previously been reported on the surface of monocytes in hypercholesterolemia and in mature macrophages in atherosclerotic lesions [37,38]. This association between CD11b and CD11c seems to induce IL-1β production and enhance macrophage adhesion to the intima wall [39,40].

**Figure 6:**
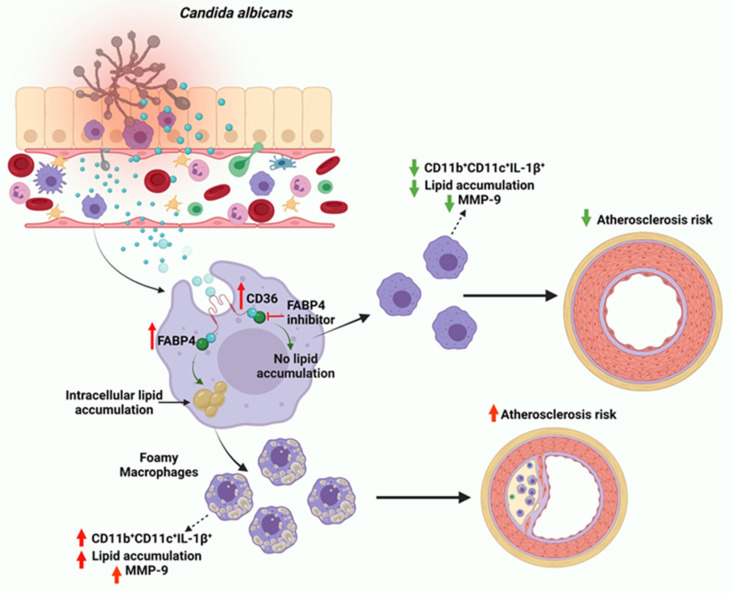
Proposed model of heat-killed *Candida albicans* (HKCA)-induced macrophage foaming. Stimulation with HKCA upregulates lipid accumulation through the elevation of CD36 and FABP4. Both proteins increase the levels of intracellular lipids that induce the macrophage proinflammatory response and MMP-9 secretion, a phenomenon known to be associated with an increased risk of atherosclerosis. The deficiency of FABP4 modulates this phenomenon, thereby preventing both lipid accumulation and macrophage inflammation. These data support the hypothesis that FABP4 can serve as a therapeutic target to alleviate *C. albicans*-induced cell foaming and inflammation.

It is still unclear how pathogen-induced inflammation within foam cells is initiated. However, recent immunometabolism studies have shown that changes in the metabolic activity of macrophages, especially those that govern lipid modulation, were found to influence macrophage activation status and function [41]. Previous work in tuberculosis infection models, a setting that induces macrophage foaming and proinflammatory responses, upregulated the expression of nuclear PPARγ [42]. Other researchers have documented this effect by identifying the role of PPARγ in triggering lipid build-up in foamed cells [42,43]. Similarly, we found that stimulation with HKCA upregulated PPARγ gene expression in macrophages. Our results showed that HKCA induces the production of the fat uptake proteins CD36 and FABP4. Both proteins have been documented to facilitate foam cell formation in atherosclerosis [27,44,45]. However, we observed that FABP4 expression was higher than CD36 expression in macrophages treated with HKCA. It has been reported that FABP4 is a lipid chaperone that binds fatty acid precursors and facilitates their delivery to PPARγ [44,46]. It undergoes conformational changes in response to ligand interactions, adopting a closed/active conformation that enables nuclear translocation and consequent PPARγ activation [47]. This process occurs in the intracellular milieu of the cell, and the binding affinity of the precursor depends upon the concentration of available FABP4 [45]. Therefore, we questioned the outcome of inhibiting FABP4 during HKCA stimulation. We found that the inhibition of FABP4 by BMS309403, or siRNA-mediated knockdown, abolished the macrophage fat accumulation observed in HKCA-stimulated cells. This process subsequently prevented proinflammatory cytokine production. FABP4 inhibition was significantly correlated with the downregulation of MMP-9, a major contributor to atherosclerotic plaque instability and disease progression [48]. MMP-9, also known as gelatinase B or type IV collagenase, has been linked to atherosclerosis development [49]. Increased circulating levels of MMP-9 have been reported in type 2 diabetes patients with coronary artery disease [50]. The association between MMP-9 plasma levels and the documented cardiovascular risk factors is believed to be triggered by circulatory MMP-9, promoting the transmigration of pro-inflammatory monocytes in the plasma across the endothelium and causing distribution in the basement membrane surrounding the endothelial cells [51,52]. One study demonstrated the effect of different cell fragments of several *Candida* species on converting host pro-MMP-9 to its active form, which contributes to tissue inflammation [53]. Our results showed that HKCA induces MMP-9 production by macrophages, along with lipid accumulation via FABP4. Overall, the thematic illustration supporting our data is shown below (Figure 6). 

However, our study involves one limitation. We believe that using live *C. albicans* could provide much potent inflammatory response along with foaming. Due to institutional policy restrictions, we could not use live *C. albicans*.

## 5. Conclusions

Our study shows that (1) HKCA stimulation increased macrophage foaming by upregulating intracellular lipid accumulation and inflammatory responses. (2) HKCA stimulation modulated the lipid uptake protein CD36 and lipid transporter protein FABP4, and (3) inhibition of or deficiency in FABP4 in macrophages prevented HKCA-induced lipid accumulation and inflammation. FABP4 inhibition also abolished HKCA-induced MMP-9 production and secretion. Therefore, FABP4 inhibition could be a potential therapeutic target in treating *C. albicans*-induced atherosclerosis.

## Data Availability

Not applicable.

## Supplementary Materials

The following are available online at https://www.mdpi.com/article/10.3390/biomedicines9111567/s1, Figure S1: Stimulation with Heat-Killed C. albicans (HKCA) does not induces tumour necrosis factor (TNF-α) or Monocyte chemoattractant protein-1 (MCP-1) secretion in macrophage. Figure S2: Inhibition of CD36 prevent Heat- killed Candida Albicans (HKCA) induced macrophage foaming. Figure S3: Inhibition of CD36 prevent Heat- killed Candida Albican (HKCA) induces MMP-9 protein expression.
