# Supplementary material for: Candida albicans Induces Foaming and Inflammation in Macrophages through FABP4: Its Implication for Atherosclerosis
Source: Biomedicines. 2021 Oct 29;9(11):1567. doi: 10.3390/biomedicines9111567 (PMC8615257; doi:10.3390/biomedicines9111567)
Supplement: Supplementary file 1 [file biomedicines-09-01567-s001.zip › biomedicines-1338037-supplementary.pdf]

Supplementary Figure S1:

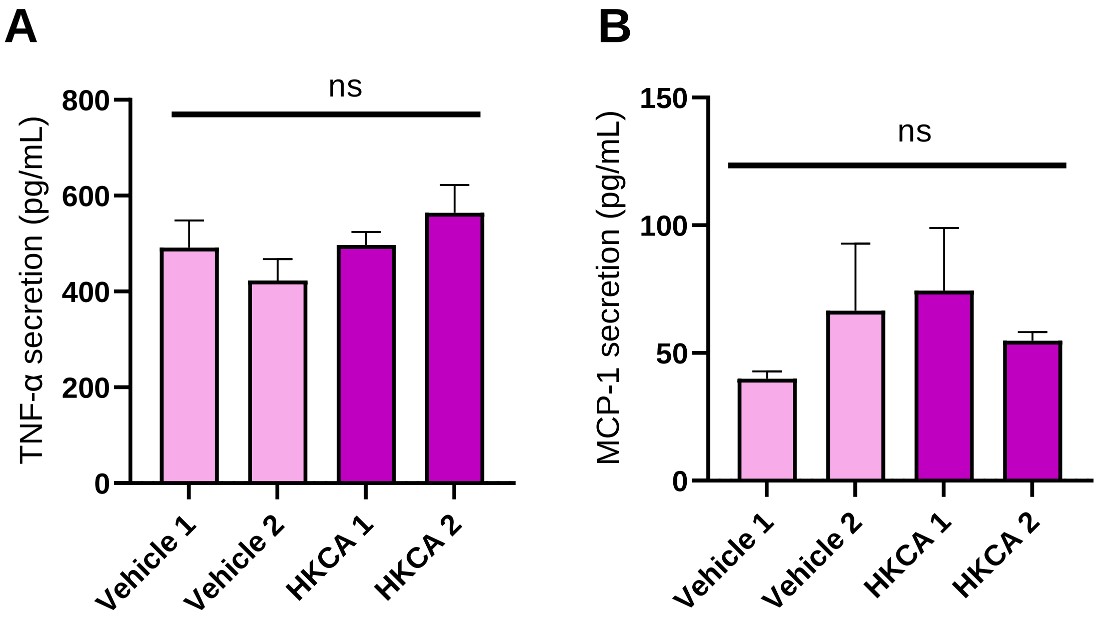

Supplementary Figure S1. Stimulation with Heat-Killed C. albicans (HKCA) does not induces tumour necrosis factor (TNF-α) or Monocyte chemoattractant protein-1 (MCP-1) secretion in macrophage . Differentiated human THP-1 macrophages were stimulated overnight with HKCA. 1, 2 indicate biological replicas (A) TNF-α and (B) MCP-1 protein secretion was measured in media by enzyme-linked immunosorbent assay (ELISA). The results obtained from three independent experiments are shown. All data are expressed as mean ± SEM values (n=3). ns: non-significant.

Supplementary Figure S2:

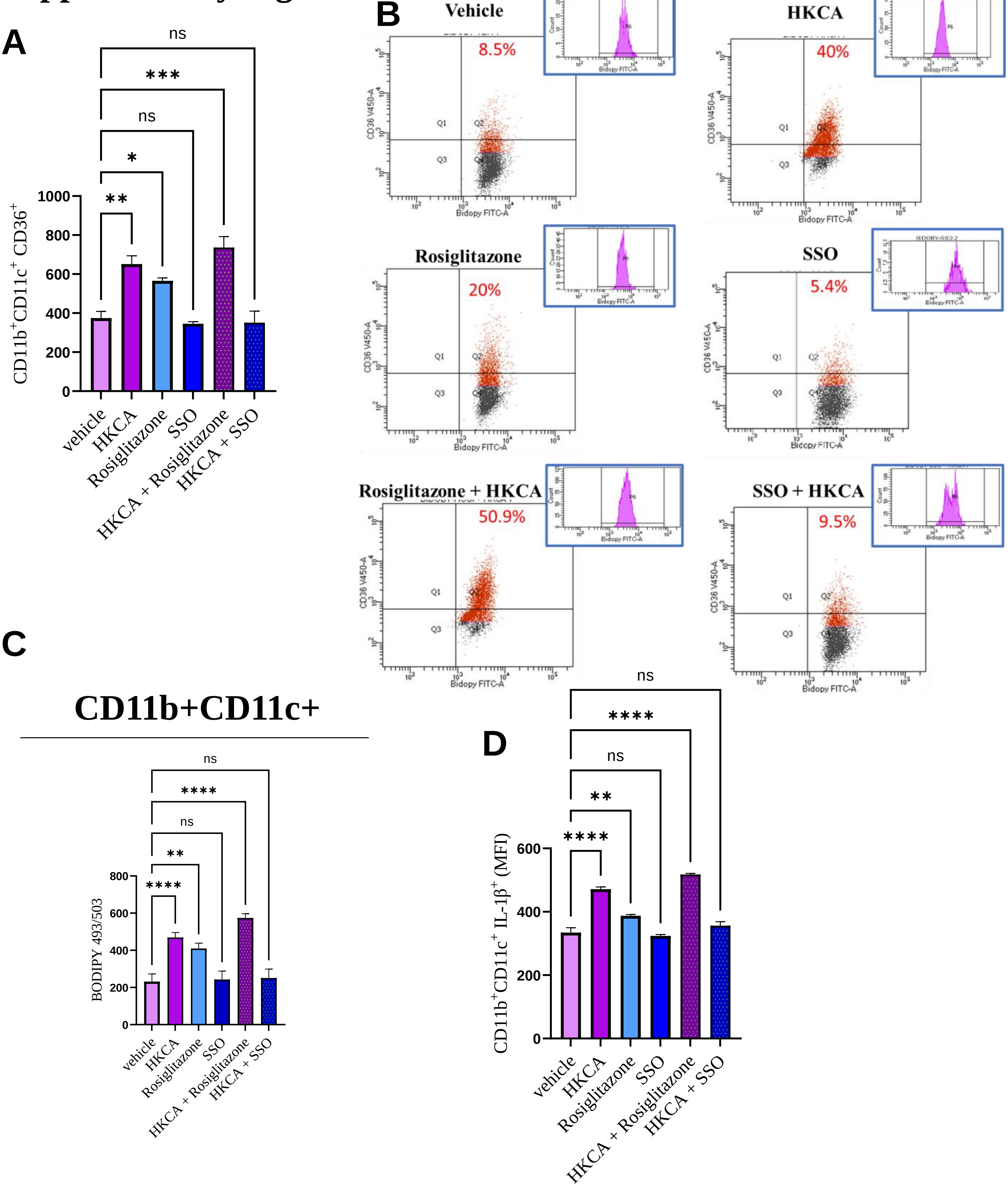

Supplementary Figure S2. Inhibition of CD36 prevent Heat- killed Candida Albicans (HKCA) induced macrophage foaming. Differentiated human THP-1 macrophages were treated with cluster of differentiation 36 (CD36) agonist (1.5μM Rosiglitazone) or 250μM CD36 inhibitor Sulfosuccinimidyl oleate (SSO) for one hour. Cells were then stimulated with HKCA overnight and the expression of CD11b , CD11c , CD36, IL-1b and BODIPY were assessed by flowcytometry. (A) Mean fluorescence intensity (MFI) of $CD11b^{+}CD11c^{+}CD36^{+}$ expression (B) percentage of cells expressing CD36 $^{+}$ $BODIPY^{+}$ with similar result presented in histogram. (C) Total expression of BODIPY 493/503 (D) Mean fluorescence intensity (MFI) of $CD11b^{+}CD11c^{+}$ IL-$1\beta^{+}$ expression The results obtained from three independent experiments are shown. All data are expressed as mean ± SEM (n = 3). *** $p \leq 0.001$, **** $p \leq 0.0001$ and ns: non-significant.

Supplementary Figure S3: Inhibition of CD36 prevent Heat- killed Candida Albican (HKCA) induces MMP-9 protein expression

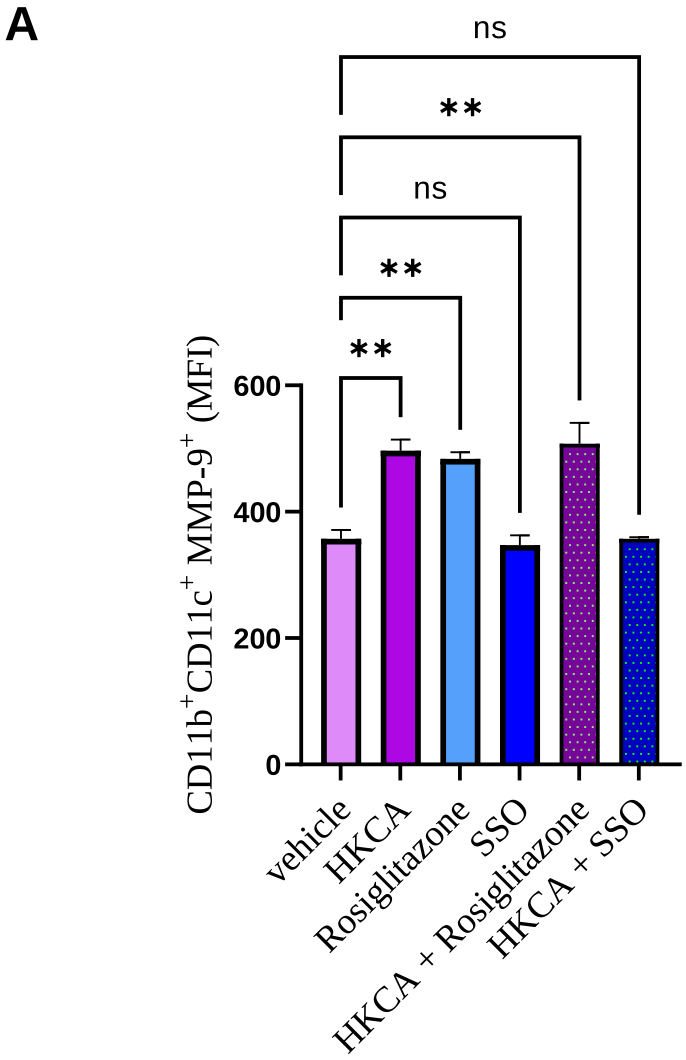

Supplementary Figure S3. Inhibition of CD36 prevent Heat- killed Candida Albicans (HKCA) induces MMP-9 protein expression. Differentiated human THP-1 macrophages were treated with cluster of differentiation 36 (CD36) agonist (1.5µM Rosiglitazone) or 250µM CD36 inhibitor Sulfosuccinimidyl oleate (SSO). Cells were then stimulated with HKCA overnight and the expression of (A) CD11b+CD11c+MMP-9+ was assessed by flowcytometry. The results obtained from three independent experiments are shown. All data are expressed as mean ± SEM values (n=3). $^{**}p \leq 0.001$, ns: non-significant.
